# Supplementary material for: A Likelihood Approach for Real-Time Calibration of Stochastic Compartmental Epidemic Models
Source: PLoS Comput Biol. 2017 Jan 17;13(1):e1005257. doi: 10.1371/journal.pcbi.1005257 (PMC5240920; doi:10.1371/journal.pcbi.1005257)
Supplement: S1 File — (TAR.GZ) [file pcbi.1005257.s014.tar.gz › HSPH_Online-SI-Revision/IPoi09/n10-severe/n10-severe_BENCH2_table-4.pdf]

1	newly 3 weeks spec	606.	733.656	{537., 877.}	29.4081	0.117249	newly 3 weeks cumul	1634.	2191.69	{1573., 2558.}	40.3606	0.153013	newly 5 weeks spec	589.	597.082	{379., 807.}	24.2729	0.110668	newly 5 weeks cumul	2883.	3464.25	{2670., 3981.}	25.9509	0.103144	newly 55 weeks cumul	5530.	5941.7	{4020., 7518.}	21.3714	0.0909746
2	newly 3 weeks spec	693.	727.746	{493., 910.}	21.2244	0.109706	newly 3 weeks cumul	1675.	2095.54	{1118., 2646.}	37.7071	0.166141	newly 5 weeks spec	711.	625.966	{329., 855.}	24.9946	0.13745	newly 5 weeks cumul	3118.	3419.29	{2456., 4168.}	21.973	0.103888	newly 57 weeks cumul	5958.	5855.83	{3758., 7656.}	17.9784	0.0844162
3	newly 3 weeks spec	672.	837.809	{563., 1066.}	32.8869	0.12737	newly 3 weeks cumul	1714.	2594.02	{2019., 3067.}	56.09	0.200713	newly 5 weeks spec	640.	543.745	{252., 888.}	33.7899	0.183201	newly 5 weeks cumul	3081.	3845.66	{2939., 4605.}	29.4026	0.113211	newly 59 weeks cumul	4699.	5074.89	{3450., 6491.}	21.8512	0.0914221
4	newly 3 weeks spec	729.	575.305	{291., 725.}	21.4131	0.153238	newly 3 weeks cumul	1838.	1554.97	{671., 2141.}	25.3472	0.180479	newly 5 weeks spec	670.	614.017	{430., 740.}	15.1606	0.0928637	newly 5 weeks cumul	3254.	2782.17	{1539., 3458.}	17.7992	0.125221	newly 54 weeks cumul	5574.	7191.81	{5576., 8283.}	30.6189	0.113623
5	newly 3 weeks spec	577.	655.877	{493., 770.}	23.4129	0.101946	newly 3 weeks cumul	1549.	1842.42	{1169., 2239.}	32.265	0.138721	newly 5 weeks spec	572.	628.015	{455., 756.}	20.4115	0.084853	newly 5 weeks cumul	2731.	3132.54	{2427., 3589.}	22.9288	0.097216	newly 54 weeks cumul	5177.	6737.66	{5225., 8219.}	33.0251	0.12093
6	newly 3 weeks spec	667.	527.781	{268., 735.}	25.2999	0.155352	newly 3 weeks cumul	1499.	1313.47	{561., 2086.}	31.3647	0.179078	newly 5 weeks spec	615.	642.766	{485., 764.}	17.5381	0.0806874	newly 5 weeks cumul	2818.	2553.89	{1387., 3412.}	22.002	0.121908	newly 55 weeks cumul	4913.	7691.14	{6867., 8368.}	56.5467	0.192935
7	newly 3 weeks spec	404.	727.828	{532., 885.}	81.4705	0.252432	newly 3 weeks cumul	1214.	2220.66	{1894., 2537.}	85.0649	0.266522	newly 5 weeks spec	360.	571.203	{327., 792.}	62.6085	0.196675	newly 5 weeks cumul	2017.	3448.92	{2617., 4032.}	72.0044	0.230945	newly 54 weeks cumul	3462.	5588.05	{3699., 7044.}	62.226	0.198463
8	newly 3 weeks spec	687.	919.078	{692., 1063.}	39.3958	0.149871	newly 3 weeks cumul	1740.	2380.27	{1343., 2990.}	46.6734	0.1754	newly 5 weeks spec	686.	880.234	{723., 1020.}	29.9913	0.112579	newly 5 weeks cumul	3148.	4192.57	{3260., 4685.}	37.164	0.140304	newly 58 weeks cumul	4943.	7895.52	{7040., 8794.}	59.7313	0.201824
9	newly 3 weeks spec	655.	760.367	{455., 1007.}	30.3868	0.122446	newly 3 weeks cumul	1488.	2081.2	{1075., 2838.}	51.6174	0.184181	newly 5 weeks spec	787.	593.3	{272., 846.}	29.6103	0.177895	newly 5 weeks cumul	3066.	3384.	{2481., 4031.}	21.0698	0.0872385	newly 62 weeks cumul	5852.	4658.57	{3399., 5544.}	20.3936	0.105259
10	newly 3 weeks spec	791.	1172.92	{726., 1526.}	53.9652	0.183162	newly 3 weeks cumul	2024.	3354.73	{1951., 4268.}	73.5705	0.241705	newly 5 weeks spec	740.	771.271	{274., 1375.}	43.9922	0.206755	newly 5 weeks cumul	3581.	5118.61	{3942., 6033.}	44.9865	0.157904	newly 62 weeks cumul	5507.	6496.61	{4515., 7985.}	25.3702	0.0993686
11	newly 3 weeks spec	764.	791.577	{567., 992.}	17.1416	0.0735393	newly 3 weeks cumul	1667.	2357.85	{1624., 2819.}	45.2771	0.160247	newly 5 weeks spec	893.	506.885	{264., 787.}	43.238	0.280683	newly 5 weeks cumul	3478.	3536.81	{2921., 4116.}	12.035	0.0523317	newly 61 weeks cumul	4839.	4482.42	{3462., 5353.}	13.2457	0.0633582
12	newly 3 weeks spec	676.	790.	{567., 992.}	24.8215	0.0991218	newly 3 weeks cumul	1411.	2357.	{1523., 2878.}	68.8971	0.221317	newly 5 weeks spec	750.	519.793	{245., 834.}	34.5011	0.218233	newly 5 weeks cumul	2924.	3560.66	{2921., 4205.}	25.2288	0.0971374	newly 61 weeks cumul	5377.	4554.41	{3424., 5476.}	16.9746	0.0888866
13	newly 3 weeks spec	785.	692.968	{350., 931.}	24.9106	0.179106	newly 3 weeks cumul	1843.	1753.74	{687., 2636.}	35.5941	0.233215	newly 5 weeks spec	871.	761.468	{592., 916.}	15.7457	0.106151	newly 5 weeks cumul	3619.	3268.97	{1874., 4228.}	21.9326	0.152478	newly 58 weeks cumul	6232.	7864.18	{6813., 8898.}	27.3104	0.103471
14	newly 3 weeks spec	790.	1012.41	{653., 1184.}	36.0581	0.139205	newly 3 weeks cumul	2006.	2443.45	{1298., 3341.}	37.0333	0.149322	newly 5 weeks spec	648.	1037.96	{824., 1188.}	60.1787	0.200346	newly 5 weeks cumul	3522.	4566.01	{3380., 5216.}	34.0072	0.127953	newly 60 weeks cumul	4964.	8490.7	{7747., 9085.}	71.0456	0.232302
15	newly 3 weeks spec	973.	705.52	{285., 1254.}	42.7749	0.259322	newly 3 weeks cumul	2131.	2874.68	{1999., 3618.}	37.1919	0.131475	newly 5 weeks spec	967.	212.8	{47., 553.}	77.9938	0.835798	newly 5 weeks cumul	4148.	3509.44	{2234., 4584.}	20.7541	0.107438	newly 64 weeks cumul	6236.	3683.4	{2244., 4992.}	40.9333	0.244551
16	newly 3 weeks spec	860.	471.277	{197., 836.}	50.8115	0.345305	newly 3 weeks cumul	2325.	2078.34	{1188., 3301.}	27.7346	0.141345	newly 5 weeks spec	767.	187.532	{64., 378.}	75.8662	0.72226	newly 5 weeks cumul	3984.	2565.89	{1413., 4111.}	39.9209	0.246062	newly 60 weeks cumul	5671.	2815.19	{1501., 4502.}	50.9884	0.348749
17	newly 3 weeks spec	584.	439.235	{164., 787.}	44.4601	0.271454	newly 3 weeks cumul	1339.	1717.32	{845., 2518.}	45.5805	0.168885	newly 5 weeks spec	581.	200.147	{36., 469.}	67.0092	0.645958	newly 5 weeks cumul	2523.	2225.85	{937., 3517.}	36.8014	0.195421	newly 60 weeks cumul	5901.	2509.35	{1039., 4325.}	57.4758	0.429775
18	newly 3 weeks spec	574.	813.364	{602., 971.}	42.8967	0.150267	newly 3 weeks cumul	1527.	2331.14	{1765., 2820.}	54.6556	0.185642	newly 5 weeks spec	639.	667.659	{360., 960.}	30.2568	0.133492	newly 5 weeks cumul	2806.	3770.34	{3064., 4364.}	35.0264	0.127563	newly 58 weeks cumul	5958.	5650.3	{3786., 7103.}	16.2543	0.0770495
19	newly 3 weeks spec	718.	645.339	{302., 831.}	20.7678	0.162784	newly 3 weeks cumul	1880.	1759.58	{679., 2377.}	27.9152	0.202145	newly 5 weeks spec	617.	642.	{485., 793.}	19.8567	0.119355	newly 5 weeks cumul	3238.	3053.19	{1763., 3770.}	19.054	0.142635	newly 54 weeks cumul	5565.	7268.63	{5995., 8604.}	32.2711	0.118417
20	newly 3 weeks spec	409.	571.561	{379., 701.}	47.0124	0.17241	newly 3 weeks cumul	1135.	1654.98	{987., 2034.}	54.9919	0.199515	newly 5 weeks spec	432.	531.632	{360., 676.}	31.4368	0.120378	newly 5 weeks cumul	2027.	2752.02	{1955., 3293.}	42.1087	0.15648	newly 54 weeks cumul	5250.	5576.33	{4063., 6993.}	20.9855	0.0896417
21	newly 3 weeks spec	797.	846.089	{382., 1243.}	34.6605	0.231002	newly 3 weeks cumul	1935.	2809.51	{1922., 3570.}	53.6675	0.273226	newly 5 weeks spec	789.	411.133	{122., 809.}	54.6684	0.485959	newly 5 weeks cumul	3576.	3842.87	{2288., 4921.}	24.1505	0.194836	newly 62 weeks cumul	5739.	4373.22	{2452., 5811.}	25.3889	0.23595
22	newly 3 weeks spec	515.	317.474	{89., 545.}	45.161	0.3271	newly 3 weeks cumul	1274.	1295.05	{545., 2078.}	35.0987	0.162995	newly 5 weeks spec	507.	144.947	{10., 458.}	73.8192	0.854172	newly 5 weeks cumul	2335.	1664.	{588., 2703.}	37.8339	0.240529	newly 60 weeks cumul	4471.	1870.95	{589., 3268.}	58.1537	0.45153
23	newly 3 weeks spec	509.	690.811	{377., 1018.}	47.9742	0.165163	newly 3 weeks cumul	1486.	2382.68	{1724., 3039.}	63.0514	0.206854	newly 5 weeks spec	479.	403.83	{156., 742.}	43.4947	0.239908	newly 5 weeks cumul	2515.	3328.91	{2079., 4402.}	39.4111	0.139626	newly 59 weeks cumul	4951.	4171.7	{2350., 6054.}	26.5908	0.142526
24	newly 3 weeks spec	864.	653.208	{209., 987.}	31.3352	0.25888	newly 3 weeks cumul	1807.	1476.69	{385., 2416.}	37.6898	0.295069	newly 5 weeks spec	1016.	872.361	{561., 1057.}	16.1773	0.121367	newly 5 weeks cumul	3920.	3140.75	{1275., 4363.}	26.155	0.195635	newly 60 weeks cumul	6534.	8418.53	{7630., 9057.}	29.0855	0.109882
25	newly 3 weeks spec	883.	465.	{14., 790.}	47.3386	0.578516	newly 3 weeks cumul	1516.	1121.95	{44., 2090.}	45.0165	0.534186	newly 5 weeks spec	1165.	444.65	{40., 631.}	61.8326	0.655115	newly 5 weeks cumul	3941.	2042.4	{103., 3164.}	48.1756	0.589193	newly 63 weeks cumul	5681.	4002.6	{3296., 4659.}	29.5441	0.363852
26	newly 3 weeks spec	596.	1096.04	{688., 1352.}	87.5217	0.271453	newly 3 weeks cumul	1544.	3019.87	{1303., 3805.}	104.499	0.317652	newly 5 weeks spec	608.	765.685	{338., 1307.}	50.3381	0.187	newly 5 weeks cumul	2767.	4770.94	{3489., 5480.}	74.8846	0.242912	newly 62 weeks cumul	4667.	6217.37	{4948., 7317.}	34.892	0.126534
27	newly 3 weeks spec	646.	907.158	{582., 1174.}	44.9568	0.15766	newly 3 weeks cumul	1397.	2580.39	{1933., 3142.}	87.5617	0.269387	newly 5 weeks spec	776.	489.263	{211., 794.}	40.3554	0.267437	newly 5 weeks cumul	2980.	3785.66	{3182., 4338.}	28.9889	0.10879	newly 63 weeks cumul	6059.	4507.39	{3439., 5266.}	25.9662	0.13566
28	newly 3 weeks spec	775.	915.063	{519., 1248.}	33.3427	0.14151	newly 3 weeks cumul	1597.	1911.19	{957., 2946.}	40.5956	0.168448	newly 5 weeks spec	804.	1185.02	{1043., 1312.}	49.3917	0.174913	newly 5 weeks cumul	3259.	4221.64	{2973., 5293.}	35.4283	0.135372	newly 62 weeks cumul	5190.	8525.14	{7862., 9093.}	64.2609	0.214911
29	newly 3 weeks spec	901.	1384.74	{565., 1758.}	70.5202	0.310306	newly 3 weeks cumul	1967.	2982.6	{858., 4388.}	73.85	0.312294	newly 5 weeks spec	1020.	1227.74	{785., 1627.}	34.0164	0.191826	newly 5 weeks cumul	4131.	5651.47	{3763., 6791.}	46.6617	0.215251	newly 64 weeks cumul	5712.	8172.21	{6617., 8995.}	46.6525	0.211572
30	newly 3 weeks spec	883.	988.382	{374., 1574.}	45.0736	0.202363	newly 3 weeks cumul	1653.	1884.37	{581., 3571.}	53.8548	0.238885	newly 5 weeks spec	1189.	1386.56	{1097., 1587.}	20.9222	0.0828852	newly 5 weeks cumul	4088.	4580.93	{2748., 6245.}	29.7402	0.125675	newly 64 weeks cumul	6316.	8768.69	{8181., 9222.}	38.833	0.141743
31	newly 3 weeks spec	575.	375.696	{179., 731.}	47.0926	0.294507	newly 3 weeks cumul	1530.	1749.48	{1077., 2684.}	35.4192	0.152293	newly 5 weeks spec	506.	152.913	{31., 404.}	72.1859	0.714358	newly 5 weeks cumul	2641.	2133.61	{1290., 3531.}	33.301	0.178698	newly 61 weeks cumul	4464.	2431.04	{1374., 4280.}	46.6982	0.312012
32	newly 3 weeks spec	390.	545.178	{347., 774.}	45.4644	0.156612	newly 3 weeks cumul	1183.	1859.84	{1376., 2285.}	59.0739	0.195725	newly 5 weeks spec	341.	355.	{175., 592.}	34.956	0.149587	newly 5 weeks cumul	1958.	2664.16	{1810., 3361.}	41.127	0.146903	newly 54 weeks cumul	3806.	3693.2	{2219., 5407.}	25.1182	0.116505
33	newly 3 weeks spec	499.	625.203	{196., 793.}	43.7417	0.213503	newly 3 weeks cumul	1415.	1801.56	{450., 2375.}	50.1707	0.24714	newly 5 weeks spec	521.	589.424	{384., 792.}	30.4922	0.14167	newly 5 weeks cumul	2457.	3001.68	{1130., 3691.}	39.4735	0.191274	newly 54 weeks cumul	5031.	6774.39	{4707., 8633.}	37.7308	0.133427
34	newly 3 weeks spec	526.	754.188	{577., 877.}	44.4986	0.155964	newly 3 weeks cumul	1521.	2285.7	{1963., 2513.}	50.3318	0.17475	newly 5 weeks spec	469.	589.078	{379., 777.}	35.6243	0.131716	newly 5 weeks cumul	2539.	3559.44	{2955., 4047.}	40.8072	0.145979	newly 54 weeks cumul	4810.	5726.67	{4087., 7276.}	26.2035	0.100725
35	newly 3 weeks spec	688.	932.797	{335., 1257.}	53.7963	0.260659	newly 3 weeks cumul	1586.	2636.95	{1241., 3482.}	76.6837	0.307385	newly 5 weeks spec	769.	714.22	{188., 1122.}	35.71	0.276734	newly 5 weeks cumul	3166.	4202.22	{2082., 5325.}	45.8725	0.239939	newly 61 weeks cumul	5296.	6064.61	{2242., 8213.}	36.4599	0.231885
36	newly 3 weeks spec	502.	763.267	{533., 917.}	53.8446	0.182655	newly 3 weeks cumul	1350.	2282.35	{1790., 2647.}	71.4679	0.233703	newly 5 weeks spec	557.	602.8	{325., 879.}	31.0652	0.132184	newly 5 weeks cumul	2471.	3579.78	{2714., 4131.}	46.5041	0.163571	newly 57 weeks cumul	5430.	5647.62	{3535., 7436.}	21.6295	0.0937553
37	newly 3 weeks spec	552.	1002.7	{764., 1182.}	88.2246	0.284831	newly 3 weeks cumul	1345.	2862.49	{2234., 3402.}	120.266	0.356156	newly 5 weeks spec	542.	721.213	{452., 1054.}	43.6013	0.15639	newly 5 weeks cumul	2492.	4459.36	{3730., 5078.}	82.8916	0.267113	newly 60 weeks cumul	4762.	6324.17	{5192., 7418.}	34.761	0.12763
38	newly 3 weeks spec	663.	679.846	{427., 884.}	24.0863	0.107914	newly 3 weeks cumul	1606.	2287.12	{1794., 2783.}	42.7891	0.14861	newly 5 weeks spec	672.	377.115	{154., 686.}	46.4228	0.326945	newly 5 weeks cumul	3051.	3182.15	{2268., 3838.}	17.0335	0.0730086	newly 60 weeks cumul	6153.	3785.15	{2529., 4959.}	38.4828	0.224037
39	newly 3 weeks spec	517.	690.418	{486., 887.}	36.7716	0.130936	newly 3 weeks cumul	1361.	2122.91	{1489., 2578.}	60.5718	0.20384	newly 5 weeks spec	517.	546.055	{268., 867.}	35.2453	0.151991	newly 5 weeks cumul	2422.	3304.33	{2515., 4104.}	37.7164	0.134155	newly 56 weeks cumul	5258.	5204.4	{3217., 6984.}	23.422	0.10576
40	newly 3 weeks spec	579.	746.404	{584., 871.}	33.4939	0.129644	newly 3 weeks cumul	1495.	2228.61	{1841., 2520.}	51.9932	0.188401	newly 5 weeks spec	560.	592.86	{355., 814.}	26.7701	0.117695	newly 5 weeks cumul	2700.	3513.3	{2973., 3986.}	33.5257	0.127962	newly 56 weeks cumul	5592.	5747.37	{3948., 7166.}	18.7655	0.0835227
41	newly 3 weeks spec	748.	1052.88	{225., 1499.}	66.9619	0.3431	newly 3 weeks cumul	1534.	2684.35	{324., 3918.}	101.157	0.441915	newly 5 weeks spec	1008.	770.525	{186., 1406.}	41.3368	0.35964	newly 5 weeks cumul	3552.	4431.	{1844., 5713.}	42.873	0.274007	newly 64 weeks cumul	5882.	6001.43	{4130., 7765.}	19.9027	0.194647
42	newly 3 weeks spec	764.	469.316	{145., 734.}	38.7182	0.307773	newly 3 weeks cumul	1835.	1160.39	{319., 1988.}	41.3873	0.336674	newly 5 weeks spec	835.	586.281	{294., 729.}	29.7867	0.202714	newly 5 weeks cumul	3509.	2283.25	{845., 3456.}	35.1327	0.268783	newly 55 weeks cumul	5877.	7818.19	{6802., 8425.}	33.0303	0.122556
43	newly 3 weeks spec	917.	1237.65	{695., 1582.}	44.1558	0.169748	newly 3 weeks cumul	2107.	3124.62	{1225., 4226.}	65.3553	0.239145	newly 5 weeks spec	846.	924.655	{483., 1540.}	36.5571	0.152611	newly 5 weeks cumul	3927.	5199.95	{3908., 6023.}	35.8645	0.137234	newly 63 weeks cumul	5868.	6838.45	{5717., 7609.}	18.2404	0.0718808
44	newly 3 weeks spec	531.	756.5	{403., 948.}	52.0657	0.189053	newly 3 weeks cumul	1403.	1970.48	{835., 2720.}	55.8903	0.206886	newly 5 weeks spec	507.	779.359	{574., 923.}	56.0867	0.189849	newly 5 weeks cumul	2442.	3538.73	{2187., 4217.}	51.3238	0.182294	newly 57 weeks cumul	4771.	7399.39	{6095., 8437.}	55.4735	0.187876
45	newly 3 weeks spec	842.	1029.23	{659., 1200.}	30.8347	0.122636	newly 3 weeks cumul	1994.	2512.23	{1206., 3335.}	40.2622	0.158071	newly 5 weeks spec	777.	1029.77	{826., 1177.}	34.7968	0.127705	newly 5 weeks cumul	3630.	4636.36	{3324., 5202.}	31.9847	0.121011	newly 60 weeks cumul	5942.	8281.24	{7482., 8930.}	39.3679	0.142737
46	newly 3 weeks spec	429.	674.467	{345., 926.}	62.7506	0.200598	newly 3 weeks cumul	1207.	2239.3	{1615., 2797.}	85.5261	0.261378	newly 5 weeks spec	442.	391.133	{134., 756.}	43.3484	0.230942	newly 5 weeks cumul	2135.	3167.8	{2056., 3930.}	50.6979	0.170961	newly 59 weeks cumul	4374.	3886.5	{2299., 5356.}	22.8357	0.116397
47	newly 3 weeks spec	432.	553.156	{196., 870.}	52.3944	0.203949	newly 3 weeks cumul	1154.	1969.66	{998., 2599.}	76.9037	0.241875	newly 5 weeks spec	487.	286.188	{75., 712.}	58.3547	0.422956	newly 5 weeks cumul	2160.	2675.63	{1209., 3712.}	40.625	0.160689	newly 60 weeks cumul	4498.	3141.09	{1273., 4842.}	34.4674	0.215934
48	newly 3 weeks spec	691.	552.743	{226., 817.}	29.3736	0.195329	newly 3 weeks cumul	1624.	1305.07	{474., 2139.}	35.9456	0.235272	newly 5 weeks spec	702.	734.586	{518., 881.}	19.4282	0.101917	newly 5 weeks cumul	3044.	2695.67	{1410., 3762.}	26.4901	0.160539	newly 57 weeks cumul	6315.	8314.27	{7661., 8747.}	31.6591	0.118827
49	newly 3 weeks spec	507.	750.087	{330., 1024.}	62.1559	0.22344	newly 3 weeks cumul	1374.	2261.7	{786., 2870.}	77.9824	0.267345	newly 5 weeks spec	548.	579.696	{329., 934.}	37.0676	0.178321	newly 5 weeks cumul	2534.	3519.8	{1928., 4440.}	49.728	0.182802	newly 59 weeks cumul	4591.	5218.11	{3722., 7057.}	26.7551	0.112541
50	newly 3 weeks spec	1068.	669.935	{289., 1061.}	43.7054	0.28573	newly 3 weeks cumul	2428.	3184.06	{2156., 4107.}	39.7938	0.148152	newly 5 weeks spec	913.	168.548	{39., 227.}	85.0299	0.93125	newly 5 weeks cumul	4440.	3687.71	{2311., 4887.}	22.2268	0.116919	newly 64 weeks cumul	6234.	3836.1	{2312., 5318.}	38.4649	0.22758
